# Efficacy of different urinary uric acid indicators in patients with chronic kidney disease

**DOI:** 10.1186/s12882-020-01953-z

**Published:** 2020-07-22

**Authors:** Haochen Guan, Yuqi Zheng, Xun Zhou, Ying Xu, Chensheng Fu, Jing Xiao, Zhibin Ye

**Affiliations:** 1 Department of Nephrology, Huadong Hospital affiliated to Fudan University, No. 221 West Yan’an Road, Shanghai, 200040 P.R. China; 2 Shanghai Key Laboratory of Clinical Geriatric Medicine, No. 221 West Yan’an Road, Shanghai, 200040 P.R. China

**Keywords:** Chronic kidney disease, Serum uric acid, Urinary uric acid indicators, Renal function

## Abstract

**Background:**

Mounting studies have shown that hyperuricemia is related to kidney diseases through multiple ways. However, the application of urinary uric acid indicators in patients with reduced renal function is not clear. In this study, we aim to determine the effects of renal function on various indicators reflecting uric acid levels in patients with chronic kidney disease (CKD).

**Methods:**

Anthropometric and biochemical examinations were performed in 625 patients with CKD recruited from Dept of Nephrology of Huadong hospital affiliated to Fudan University. Multiple regression analyses were used to study correlations of the estimated glomerular filtration rate (eGFR) with serum uric acid (SUA) and renal handling of uric acid. For further study, smooth curve plots and threshold effect analyses were applied to clarify associations between renal function and uric acid levels.

**Results:**

The nonlinear relationships were observed between eGFR and urinary uric acid indicators. The obvious inflection points were observed in smooth curve fitting of eGFR and fractional excretion of uric acid (FEur), excretion of uric acid per volume of glomerular filtration (EurGF). In subsequent analyses where levels of eGFR< 15 mL/min/1.73m^2^ were dichotomized (CKD5a/CKD5b), patients in the CKD5a showed higher levels of FEur and EurGF while lower levels of urinary uric acid excretion (UUA), clearance of uric acid (Cur) and glomerular filtration load of uric acid (FLur) compared with CKD5b group (all *P* < 0.05). And there was no significant difference of SUA levels between two groups. On the other hand, when eGFR< 109.9 ml/min/1.73 m^2^ and 89.1 ml/min/1.73 m^2^, the resultant curves exhibited approximately linear associations of eGFR with Cur and FLur respectively.

**Conclusion:**

FEur and EurGF showed significantly compensatory increases with decreased renal function. And extra-renal uric acid excretion may play a compensatory role in patients with severe renal impairment to maintain SUA levels. Moreover, Cur and FLur may be more reliable indicators of classification for hyperuricemia in CKD patients.

## Background

Hyperuricemia is a disease characterized by abnormal elevation of serum uric acid (SUA), defined as SUA level ≥ 6.8 mg/dL based on the limit of urate solubility, which is closely related to the occurrence and development of renal diseases [1, 2]. It has demonstrated that hyperuricemia is an independent risk factor for the decline in renal function, and the incidence of hyperuricemia increases with the progression of estimated glomerular filtration rate (eGFR) staging [3–5]. Regardless of which is cause or consequence, the correlation between chronic kidney disease (CKD) and hyperuricemia became apparent [6]. Previous studies revealed the phenomenon that apart from excessive production of uric acid more than 90% of hyperuricemia is caused by the inefficient capacity of renal to clear uric acid [7]. It was believed that renal handling of uric acid mainly relied on absorption, secretion and reabsorption of renal tubules. However, a cross-sectional study revealed that glomerular function played a more important role in regulating uric acid homeostasis than previously thought [8]. This study showed that the prevalence of gout was 2.9% among those with normal GFR while 24% among those with GFR<60 ml/min/1.73 m^2^ and the prevalence of gout increased by about 2–3 times for every 30 ml/min/1.73 m^2^ decrease in GFR. In addition, it has been proved that multiple uric acid transporters are expressed at the apical and basolateral membranes of proximal tubule which coupled with numerous solutes to regulate uric acid influx and efflux. Due to the abnormal activation of renin-angiotensin-aldosterone system (RAAS) and parathyroid hormone (PTH) of CKD, ANG II and PTH stimulate the coupled entry of Na + and lactate which in turn increase urate/lactate exchange across urate transporter 1 (URAT1) causing reduced uric acid excretion [9]. Therefore, the reduced glomerular filtration and deregulated RAAS and PTH in maintaining the balance of uric acid in CKD must not be ignored. According to different combinations of urinary uric acid excretion (UUA), clearance of uric acid (Cur) and fractional excretion of uric acid (FEur), hyperuricemia can be divided into three categories: urate overproduction, decreased uric acid excretion and combined mechanism which is helpful to instruct application of urate-lowering drugs reasonably and clarify the metabolic status of uric acid in patients [10]. However, the current classification of hyperuricemia did not take renal function into account, which may affect the accurate assessment of uric acid excretion in CKD patients. As we know, the specific mechanism underlying how renal function affects uric acid excretion in the population with CKD is rarely studied. Thus, this study aims to investigate the efficacy of different urinary uric acid indicators in patients with CKD and determine the excretion of uric acid in CKD patients.

In clinical practice, the most common method to classify hyperuricemic patients is measuring urinary uric acid output per day to evaluate whether underexcretion of uric acid or not. Patients with UUA > 800 mg/d on a regular diet suggests overproduction of uric acid as the etiology [11]. However, the traditional indicator of uric acid excretion such as UUA is easily affected by many factors (for example, SUA, urine volume, serum creatinine, gender, weight) and cannot accurately reflect the excretion of uric acid of renal tubules. Perez-Ruiz [12] found that even patients with gout showing apparent high uric acid output per day showed lower Cur than controls through a case–control study, indicating that relative, low-grade underexcretion of uric acid actually. And some new parameters of uric acid are more persuasive and less to be distributed such as Cur, FEur, glomerular filtration load of uric acid (FLur), excretion of uric acid per volume of glomerular filtration (EurGF). Therefore, we analyze these indicators to confirm which parameter is superior to assess the excretion of uric acid in CKD patients.

## Methods

### Study population and sample collection

A total of 625 consecutive patients with CKD from 2015 to 2018 hospitalized in Department of Nephrology, Huadong hospital affiliated to Fudan University (Shanghai, P.R.China) were included in this study for analysis. Clinical indicators of patients needed to be stable during the study period and demanding normal purine diet without alcohol or rich fructose food for 5 days before the study. Patients were excluded according to the following exclusion criteria: taking medications known to affect renal handling of uric acid (such as aspirin, anti-tuberculosis drugs, immunosuppressive agents, diuretics, losartan, metformin) and uric acid-lowering agents (such as allopurinol, febuxostat, benzbromarone) in the previous 2 weeks; acute kidney injury, kidney transplantation, dialysis; a history of hereditary hyperuricemia; severe heart, lung or liver dysfunction, infection and tumor.

### Clinical measurements

The clinical data were extracted from the medical records of 625 CKD patients. Fasting blood samples were drawn in the morning and all patients underwent renal function tests using 24-h urine collection while on an unrestricted diet (avoiding excessive purine and fructose intake and with avoidance of alcohol). All samples were immediately sent to the laboratory for the biochemical analysis including hemoglobin (HB), C-reactive protein (CRP), hemoglobin A1c (HbA1c), albumin (ALB), low-density lipoprotein (LDL), high-density lipoprotein (HDL), total cholesterol (TC), triglycerides (TG), blood urea nitrogen (BUN), serum creatinine (Scr), serum uric acid (SUA).

Body mass index (BMI) was calculated from formula BMI = weight / height^2^, expressed in kg/ m^2^. Urinary uric acid (UUA) represented twenty-four hours uric acid was calculated as uric acid concentration × 24-h urinary volume (24-h UV). Urinary creatinine (Ucr) represented twenty-four hours urinary creatinine calculated as urinary creatinine concentration × 24-h UV. Clearance of creatinine (Ccr) was calculated as urinary creatinine concentration × 24-h UV / Scr, expressed in ml/min. The formula of clearance of uric acid (Cur) was urinary uric acid concentration × 24-h UV / SUA, expressed in ml/min. Glomerular filtration load of uric acid (FLur) was calculated as Ccr × SUA, expressed in μmol/min. Fractional excretion of uric acid (FEur) was calculated as (UUA × Scr) / (SUA × Ucr) × 100, expressed as percentage. Excretion of uric acid per volume of glomerular filtration (EurGF) was calculated as (UUA × Scr) / Ucr, expressed in μmol/L. The estimated glomerular filtration rate (eGFR) (mL/min/1.73m^2^), an indicator of renal function, was calculated using the Chronic Kidney Disease Epidemiology Collaboration (CKD-EPI) formula.

### Grouping criteria

According to the Kidney Disease: Improving Global Outcomes CKD guidelines, we divided patients into 5 groups based on levels of eGFR, eGFR categories were defined as follows: ≥90 mL/min/1.73m^2^ (CKD1), 60-89 mL/min/1.73m^2^ (CKD2), 30–59 mL/min/1.73m^2^ (CKD3), 15–29 mL/min/1.73m^2^ (CKD4) and < 15 mL/min/1.73m^2^ (CKD5). According to the inflection points of FEur and EurGF, we divided patients with CKD5 into CKD5a: 7.5 mL/min/1.73m^2^ ≤ eGFR < 15 mL/min/1.73m^2^ and CKD5b: eGFR < 7.5 mL/min/1.73m^2^.

### Statistical analysis

Data are presented as mean ± standard deviation (SD), median with inter quartile range (IQR) or percentages for normally distributed continuous variables, non-normally distributed continuous variables and categorical variables, respectively. The distribution of all examined variables was assessed by Shapiro-Wilk test, histograms and probability plots. T test was used between the two groups, and One-way ANOVA was used to compare normally distributed data. We used Kruskall-Wallis test to compare non-normally distributed data and Chi-square test for categorical data. Then we applied multiple regression analyses to determine the independent associations between renal function and uric acid levels (including SUA, UUA, Cur, FLur, FEur, EurGF), with an adjustment for multiple confounding factors. And we further applied a two-piecewise linear regression model to examine the threshold effect of the eGFR on uric acid levels using a smoothing function. Likelihood ratio tests were conducted to compare the one-line linear regression model with a two-piecewise linear model. Statistical significance was set at *P* < 0.05. All statistical analysis was performed with EmpowerStats (www.empowerstats.com), the statistical package R, software SPSS 23.0 and GraphPad Prism 7.0.

## Results

### Baseline characteristics of the study population stratified by levels of eGFR

Based on the exclusion criteria, 625 CKD patients (318 males and 307 females) aged 57.9 ± 16.4 years were selected into the current study. General data and functional parameters in different groups according to levels of eGFR are shown in Table 1. The mean level of SUA was 393.8 μmol/L and the median UUA level was 2.3 mmol/24 h. And various indicators for evaluating uric acid levels (including SUA, UUA, Cur, FLur, EurGF, FEur) were significantly different in different groups. It seems to conclude that patients with worse renal function show higher levels of SUA, FEur, EurGF while lower level of UUA, Cur and FLur. Apart from uric acid indicators, other clinical parameters such as age, ratio of hypertension, ratio of diabetes, HB, CRP, ALB, LDL, HDL, TC, BUN, Scr, Ucr, and Ccr were all significantly different among groups.

**Table 1:** Baseline characteristics of the study population stratified by levels of eGFR

Variables	Overall (*n* = 625)	CKD1 (*n* = 130)	CKD2 (*n* = 164)	CKD3 (*n* = 147)	CKD4 (*n* = 86)	CKD5 (*n* = 97)	*P* value
Male gender (%)	50.9	50.8	56.7	45.6	48.8	50.5	0.4021
Age (years)	57.9 ± 16.4	46.2 ± 14.9	56.4 ± 14.7	62.4 ± 15.4	63.8 ± 15.4	63.9 ± 14.8	**< 0.001**
BMI (kg/m^2^)	24.3 ± 3.9	23.7 ± 3.8	24.7 ± 4.4	24.7 ± 3.5	24.2 ± 3.7	24.1 ± 4.0	0.204
Hypertension (%)	66.8	32.1	61.3	76.2	83.6	83.8	**< 0.0001**
Diabetes (%)	32	19.2	29	38.1	29.5	43.8	**0.0118**
HB (g/L)	115.9 ± 24.4	128.6 ± 18.3	131.1 ± 19.2	122.3 ± 17.7	102.7 ± 17.9	90.3 ± 18.7	**< 0.001**
CRP (mg/L)	3.7 (1.1–8.2)	2.8 (0.8–6.2)	3.2 (1.0–6.5)	3.8 (1.1–9.5)	4.7 (1.2–9.0)	6.0 (1.8–12.2)	**< 0.001**
HbA1C (%)	6.0 ± 1.1	6.0 ± 1.5	6.0 ± 0.9	6.2 ± 1.1	6.1 ± 1.0	5.9 ± 1.0	0.637
ALB (g/L)	37.8 ± 7.6	38.2 ± 7.6	38.8 ± 9.7	38.6 ± 6.4	36.7 ± 6.6	35.5 ± 5.5	**0.004**
LDL (mmol/L)	2.8 ± 1.1	3.1 ± 1.2	3.0 ± 1.3	2.7 ± 0.9	2.7 ± 1.0	2.4 ± 0.8	**< 0.001**
HDL (mmol/L)	1.3 ± 0.4	1.4 ± 0.3	1.3 ± 0.4	1.3 ± 0.4	1.3 ± 0.3	1.2 ± 0.3	**0.003**
TC (mmol/L)	4.9 ± 1.5	5.1 ± 1.7	5.1 ± 1.7	4.7 ± 1.4	4.9 ± 1.5	4.4 ± 1.1	**0.002**
TG (mmol/L)	1.9 ± 1.4	1.9 ± 1.3	2.0 ± 1.8	1.9 ± 1.1	2.1 ± 1.7	1.6 ± 0.8	0.188
BUN (mmol/L)	10.5 ± 8.2	4.9 ± 1.3	6.6 ± 5.1	8.5 ± 2.7	13.2 ± 4.6	24.9 ± 8.4	**< 0.001**
Scr (μmol/L)	199.0 ± 218.6	63.5 ± 11.5	89.8 ± 15.6	134.9 ± 28.5	227.5 ± 43.7	631.6 ± 250.3	**< 0.001**
SUA (μmol/L)	393.8 ± 110.7	329.1 ± 89.3	367.1 ± 102.2	423.6 ± 96.5	414.8 ± 106.6	461.0 ± 116.7	**< 0.001**
UUA (mmol/24 h)	2.3 (1.7–3.1)	2.9 (2.3–3.6)	2.7 (2.2–3.4)	2.3 (1.7–2.8)	1.8 (1.3–2.4)	1.4 (0.8–1.9)	**< 0.001**
Ccr (mL/min)	60.1 (26.7–93.1)	114.3 (92.9–135.6)	83.2 (69.3–101.7)	52.0 (43.0–65.2)	26.4 (20.8–35.1)	9.3 (5.9–14.3)	**< 0.001**
Cur (mL/min)	4.3 (2.7–6.0)	6.6 (4.6–8.6)	5.2 (4.2–6.9)	3.8 (3.0–4.9)	2.9 (2.2–4.4)	2.0 (1.3–2.7)	**< 0.001**
FLur (μmol/min)	22.3 (10.9–33.1)	38.2 (26.9–47.6)	30.7 (22.4–41.8)	22.2 (16.2–26.8)	11.2 (7.6–15.9)	4.1 (2.7–6.3)	**< 0.001**
EurGF (μmol/L)	26.7 (20.2–46.1)	18.0 (15.2–20.7)	22.4 (19.8–27.1)	29.2 (23.6–37.6)	50.6 (35.7–58.6)	91.2 (67.0–135.4)	**< 0.001**
FEur (%)	7.3 (5.5–11.3)	5.5 (4.3–7.1)	6.1 (5.1–7.6)	7.3 (5.8–9.2)	11.1 (7.9–15.3)	20.7 (15.6–29.7)	**< 0.001**

Normally distributed variables are presented as mean ± SD. For non-normally distributed data such as CRP, UUA, Ccr, Cur, FLur, EurGF and FEur, medians and the 25th and 75th percentiles are shown. Categorical variables are presented as percentages. *P* values are assessed with one-way ANOVA, Kruskal-Wallis or Chi-square test, as appropriate. Bold indicates statistical significance (*P* value < 0.05).

### Association of renal function with uric acid levels

We applied multiple regression models to examine association of renal function with SUA, UUA, Cur, FLur, EurGF and FEur using eGFR as a continuous variable and in categories (Table 2). When we examined eGFR as a continuous variable, we found inverse associations with SUA, FEur, EurGF (β = − 1.41, 95%CI: − 1.64,-1.17; β = − 0.15, 95%CI: − 0.16,-0.13 and β = − 0.76, 95%CI: − 0.84,-0.67, respectively) and positive correlation with UUA, Cur, FLur (β = 0.01, 95%CI: 0.01,0.02; β = 0.04, 95%CI: 0.04,0.05 and β = 0.33, 95%CI: 0.29,0.36, respectively) after adjusting for potential confounders (all *P* < 0.01). With eGFR cutoffs, patients in CKD2-CKD5 stage had higher SUA levels and lower Cur and FLur levels compared with CKD1 stage patients (all *P* < 0.01). And CKD3–5 stage patients showed lower levels of UUA and higher levels of FEur and EurGF compares with CKD1 patients (all *P* < 0.01) with the adjustment of multiple confounders.

**Table 2:** Adjusted effects of renal function on levels of uric acid

All(*n* = 625)	SUA (μmol/L)β	UUA (mmol/24 h)β	Cur (mL/min)β	FLur (μmol/min)β	FEur (%)β
	(95% CI)	(95% CI)	(95% CI)	(95% CI)	(95% CI)
Continuous eGFR (mL/min/1.73^2^)	−1.41 (− 1.64, − 1.17)**	0.01 (0.01, 0.02)**	0.04 (0.04, 0.05)**	0.33 (0.29, 0.36)**	−0.15 (− 0.16, − 0.13)**
**Clinical cutoffs**					
CKD 1 (*n* = 130)	Reference	Reference	Reference	Reference	Reference
CKD 2 (*n* = 164)	35.36 (12.13,58.59)	−0.17(−0.50, 0.17)	−1.02 (− 1.78, − 0.27)	−6.13(−9.68, −2.59)**	1.23(− 0.51, 2.96)
CKD 3 (*n*=147)	99.55 (74.95,124.16)**	−0.43(− 0.78, − 0.07)*	−2.49 (− 3.29, − 1.70)**	− 15.93(− 19.68, − 12.17)**	2.80 (0.95, 4.65)**
CKD 4 (*n* = 86)	99.08 (71.00,127.17)**	−0.87 (− 1.27, 0.46)**	−3.32 (− 4.24, − 2.41)**	− 24.80(− 29.09, − 20.52)**	6.58 (4.47, 8.69)**
CKD 5 (*n* = 97)	146.02 (118.80,173.25)**	−1.41(− 1.80, 1.01)**	−4.59 (− 5.47, − 3.70) **	− 32.01(− 36.17, − 27.85)**	17.41 (15.37, 19.46)**

Multiple regression analyses were used to estimate associations of renal function with uric acid after adjusting for age, height, weight, body surface area. Values reflect the difference and 95% CI for categories of eGFR as compared with the reference category. Values for eGFR continuously reflect a difference per mL/min/1.73m^2^ in levels of eGFR. CKD1: eGFR ≥90 mL/min/1.73m^2^, CKD2: 89 ≥ eGFR ≥60 mL/min/1.73m^2^, CKD3: 59 ≥ eGFR ≥30 mL/min/1.73m^2^, CKD4: 29 ≥ eGFR ≥15 mL/min/1.73m^2^, CKD5: eGFR < 15 mL/min/1.73 m^2^. ^*^P<0.05, ^**^P<0.01.

### Smooth curve fitting and threshold effect analysis of eGFR on uric acid levels

**Fig. 1:**
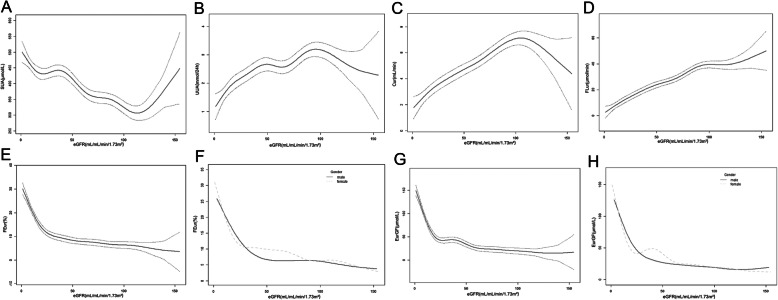
Smooth curve fitting of eGFR and uric acid levels. **a** eGFR and SUA; **b** eGFR and UUA; **c** eGFR and Cur; **d** eGFR and FLur; **e** eGFR and FEur; **f** eGFR and FEur (grouped by gender); **g** eGFR and EurGF; **h** eGFR and EurGF (grouped by gender). Solid smooth curves represent the fitting results of eGFR and dotted smooth curves represent the 95% CI of eGFR except Figure F and H which solid smooth curves represent male and dotted smooth curves represent female. The nonlinear relationships between eGFR and uric acid levels were observed after adjusting for age, height, weight, body surface area. And an obvious inflection point exists in smooth curve fitting of eGFR and FEur as well as eGFR and EurGF, and different inflection points appear according to gender grouping

Smooth curve plots were performed after the adjustment of confounding factors, and the resultant curves exhibited nonlinear associations of eGFR with SUA, UUA, Cur and FLur. In addition, two-stage changes and inflection points were observed in FEur and EurGF, and different inflection points appeared according to gender grouping (Fig. 1). The levels of FEur and EurGF increased with decreasing eGFR values up to the inflection points (15.9 mL/min/1.73m^2^ and 15.3 mL/min/1.73m^2^, respectively) with or without the adjustment of potential confounders. When the eGFR values were less than the points, the FEur and EurGF levels presented dramatical decrease (β = − 1.4, 95%CI: − 1.6,-1.2, *P* < 0.001 and β = − 7.6, 95%CI: − 8.5,-6.7, *P* < 0.001); in contrast, if the values were more than the points, the FEur and EurGF levels showed mild decrease (β = − 0.1, 95%CI: − 0.1,-0.0, *P* < 0.001 and β = − 0.4, 95%CI: − 0.5,-0.3, *P* < 0.001). The threshold effects were also analyzed based on gender grouping. The data indicated that the inflection point of FEur was 28.9 mL/min/1.73m^2^ in male patients, 8 mL/min/1.73m^2^ in female patients and the inflection point of EurGF was 20.2 mL/min/1.73m^2^ in male patients, 13.9 mL/min/1.73m^2^ in female patients after adjusting for confounders (Fig. 1 and Table 3). On the other hand, when eGFR< 109.9 ml/min/1.73 m^2^ and 89.1 ml/min/1.73 m^2^, the resultant curves exhibited approximately linear associations of eGFR with Cur and FLur respectively (Fig. 1).

**Table 3:** Threshold effect analysis of eGFR on uric acid levels using piece-wise linear regression

Model	Model I									Model II							
Various	Male			Female			Total			Male			Female			Total	
	Crude β/OR (95%CI)	*p* value		Crude β/OR (95%CI)	*p* value		Crude β/OR (95%CI)	*p* value		Crude β/OR (95%CI)	*p* value		Crude β/OR (95%CI)	*p* value		Crude β/OR (95%CI)	*p* value
**FEur (%)**									**FEur (%)**								
eGFR< 28.9	− 0.7 (− 0.8, − 0.6)	< 0.001	eGFR< 8	−4.5 (−5.6, −3.4)	< 0.001	eGFR< 15.9	− 1.4 (− 1.6, − 1.2)	< 0.001	eGFR< 28.9	− 0.7 (− 0.8, − 0.6)	< 0.001	eGFR< 8	− 4.5 (− 5.6, − 3.4)	< 0.001	eGFR< 15.9	− 1.4 (− 1.6, − 1.2)	< 0.001
eGFR≥28.9	−0.0 (− 0.1, − 0.0)	0.002	eGFR≥8	−0.1 (− 0.1, − 0.1)	< 0.001	eGFR≥15.9	−0.1 (− 0.1, − 0.0)	< 0.001	eGFR≥28.9	−0.0 (− 0.1, − 0.0)	0.002	eGFR≥8	−0.1 (− 0.1, − 0.1)	< 0.001	eGFR≥15.9	−0.1 (− 0.1, − 0.0)	< 0.001
**EurGF** **(μmol/L)**									**EurGF** **(μmol/L)**								
eGFR< 21.3	− 5.1 (− 5.6, − 4.6)	< 0.001	eGFR< 8	−21.6 (− 26.4, − 16.8)	< 0.001	eGFR< 15.3	−7.6 (− 8.5, − 6.7)	< 0.001	eGFR< 20.2	−5.3 (− 5.9, − 4.8)	< 0.001	eGFR< 13.9	−8.8 (− 10.6, − 7.0)	< 0.001	eGFR< 15.3	− 7.6 (− 8.5, − 6.7)	< 0.001
eGFR≥21.3	−0.3 (− 0.3, − 0.2)	< 0.001	eGFR≥8	− 0.5 (− 0.6, − 0.3)	< 0.001	eGFR≥15.3	−0.3 (− 0.4, − 0.3)	< 0.001	eGFR≥20.2	−0.3 (− 0.4, − 0.2)	< 0.001	eGFR≥13.9	−0.4 (− 0.6, − 0.3)	< 0.001	eGFR≥15.3	−0.4 (− 0.5, − 0.3)	< 0.001

The threshold effects were analyzed based on gender grouping. The data indicated that the inflection point of FEur and EurGF were different in male and female patients. Model I: no adjustment; Model II: adjusted for age, height, weight, body surface area.

β represents beta coefficient which refers to how many deviations change in the dependent variable rely on the change of independent variable.

### Differences of various uric acid indicators in patients with CKD5

**Fig. 2:**
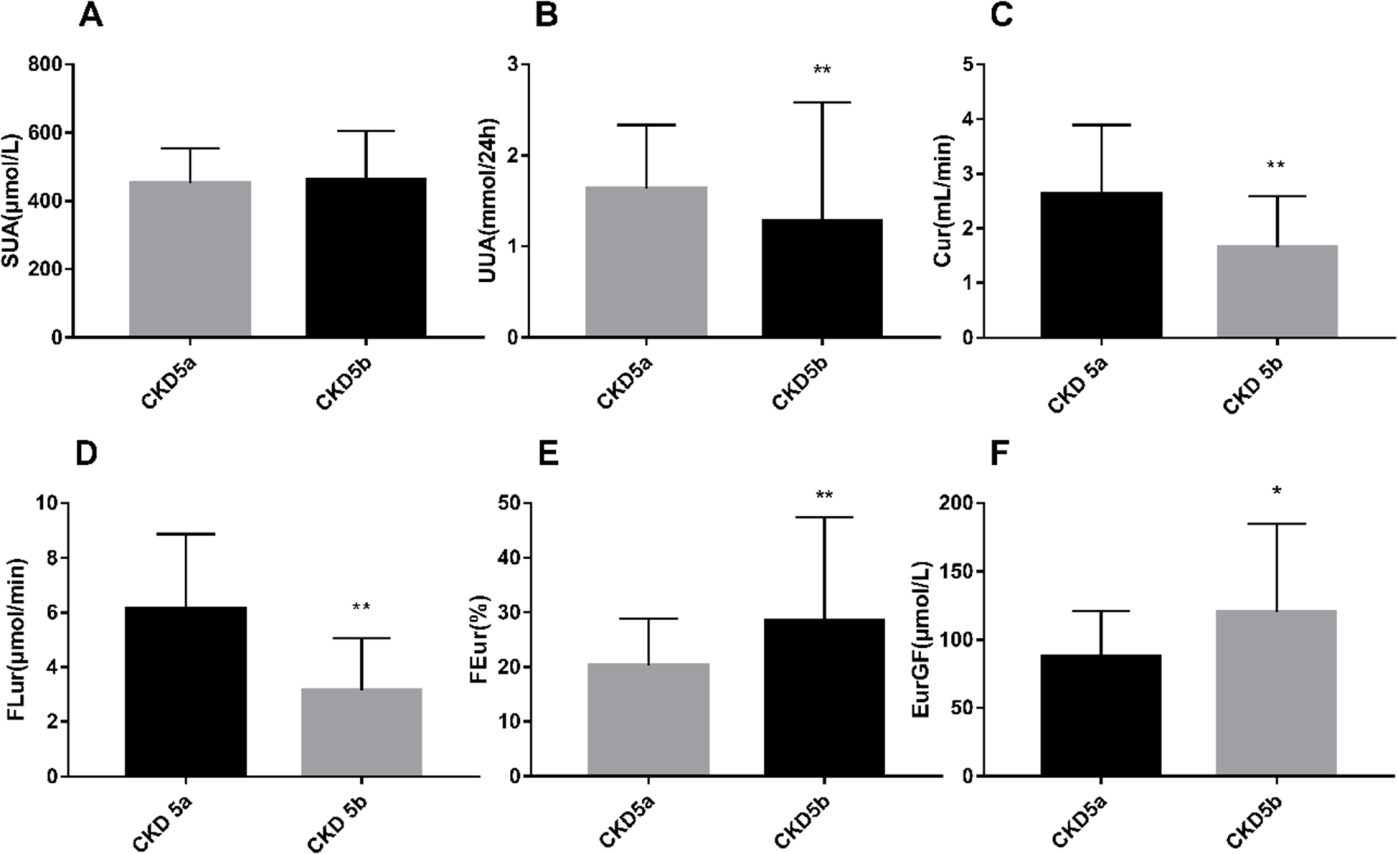
Differences of various uric acid indicators in patients with CKD5. Compared with CKD5a group, levels of FEur and EurGF were elevated significantly in CKD5b group. In contrast, levels of UUA, Cur and FLur were significantly lower in the CKD5b group compared with CKD5a group. However, no significant difference in SUA levels was observed between two groups. CKD5a: 7.5 mL/min/1.73m^2^ ≤ eGFR < 15 mL/min/1.73m^2^; CKD5b: eGFR < 7.5 mL/min/1.73m^2^. ** *P* < 0.01, * *P* < 0.05 vs. CKD 5a

According to the inflection points of FEur and EurGF, we divided all CKD5 stage patients into two groups (CKD5a: 7.5 mL/min/1.73m^2^ ≤ eGFR < 15 mL/min/1.73m^2^ and CKD5b: eGFR < 7.5 mL/min/1.73m^2^). T test was used to compare levels of SUA while non-parametric tests were applied to compare UUA, Cur, FLur, FEur and EurGF levels. Compared with CKD5a group, levels of UUA, Cur and FLur were lower in CKD5b group (*P* < 0.01; Fig. 2b, c, d). Levels of FEur and EurGF were significantly elevated in the CKD5b group vs. CKD5a group (*P* < 0.01 and *P* < 0.05 respectively; Fig. 2e, f). Interestingly, there was no significant difference of SUA levels between two groups (Fig. 2a).

## Discussion

The level of SUA is closely related to the renal excretion of uric acid. Besides, pathological change, diet, gene mutation or medicine effects may affect the excretion of uric acid [13–16]. A number of studies have shown that reduction of uric acid excretion is harmful to various systems [17], while some recent studies have demonstrated that increased uric acid excretion may be one of the causes of acute and chronic kidney disease as well. As far as we know there are few studies on the assessment of renal uric acid excretion in CKD patients. Findings from the current analysis demonstrated that inversely nonlinear associations of eGFR with SUA, FEur and EurGF, and the positively nonlinear correlation between eGFR and UUA, Cur and FLur in CKD patients. And approximately linear associations of eGFR with Cur and FLur were observed when eGFR< 109.9 ml/min/1.73 m^2^ and 89.1 ml/min/1.73 m^2^. The above results indicated that Cur and FLur are better parameters for monitoring uric acid metabolism in patients with renal dysfunction.

Although several studies have found that the association of SUA and UUA with the likelihood of CKD [18, 19] since these indicators are vulnerable to confounding factors, especially renal function, it seems insufficient to assess uric acid excretion in patients with CKD and to classify hyperuricemia based on them. And this view has been confirmed in our research. The previous study has shown that a good relationship between Cur and UUA was observed while a poor correlation between EurGF and UUA in gout patients with mild impairment of renal function. And Cur misclassified 15% gout patients and EurFG misclassified 33% patients when UUA, Cur and EurGF were used to classify gouty patients [20]. This result was compatible with ours that Cur may be a more constant indicator and reflected the intrinsic capacity for the renal handling of uric acid which was independent of renal function.

One prospective observational study found that compared with healthy controls, gout patients with eGFR > 60 ml/min/1.73 m^2^ showed higher levels of FLur and lower Cur and FEur. And changes in FLur were correlated with changes in SUA levels significantly [21]. Moreover, it should be emphasized that EurGF and UUA didn’t take SUA into account, thus omitting the effect of the FLur, and these indicators can be influenced by the FLur. In other words, the decline in CKD patient’s eGFR level is itself accompanied by a different FLur level, while EurGF or UUA may be affected under the influence of different FLur levels and weaken their value in evaluating uric acid excretion status. FLur showed a good linear relationship with eGFR suggesting its important value in reflecting uric acid excretion status in CKD patients. Although more evidence is needed to support our view, our study provides a new understanding of the efficacy of different urinary uric acid indicators in patients with CKD.

In our study, we found two-stage changes and gender-specific inflection points in FEur and EurGF which suggest these two indicators should be used cautiously in CKD patients. Patients with eGFR<7.5 mL/min/1.73 m^2^ (CKD5b) showed lower levels of UUA and higher level of FEur, EurGF while similar level of SUA compared to patients with 7.5 mL/min/1.73m^2^ ≤ eGFR< 15 mL/min/1.73m^2^ (CKD5a), suggesting there may be the compensation of residual nephrons, extra-renal excretion or presently unknown factors of uremia per se affecting the entire dynamic metabolism of uric acid. We found that FEur and EurGF dramatically increased in CKD 5 patients and speculated there is an adaptive alteration to delay the progress of kidney disease depending on eGFR. Likewise, a compensatory tubular function of creatinine and glucose excretion in CKD patients exhibited nonspecific alterations to minimize the renal disease progresses [22]. On the other hand, the reduction of renal function increased the level of EurGF which may misguide that patients with poor renal function were overproducers of the urate. Due to the declining renal function in CKD patients, urate transporters expressed at the proximal tubules cannot achieve sufficient uric acid excretion [23–25]. And more evidence has proved that some uric acid transporters also expressed at the intestinal cells which appear to maintain uric acid homeostasis especially in CKD patients [26–29]. It has been demonstrated that the extra-renal ABCG2 played a compensatory role in the setting of impaired renal function compared with renal urate transporters [30]. Thus, though FEur and EurGF eliminates the effects of other confounding factors which may reflect more on the compensatory residual kidney function in CKD patients and may not be interpreted as the real status of renal uric acid handling [31].

Though the mechanisms of renal urate handling have yet to be fully understood, urate transporter system will help us to explain how these indicators can be modified by CKD. Due to the special status of CKD, the abnormal expression of local stimulator in kidney could affect uric acid excretion, such as PTH, ANGII. With the progress of CKD, glomerular filtration function has become the main influencing factor of uric acid excretion and glomerulotubular imbalance may lead to a significant increase in FEur [32]. The EurGF of residual nephron will be correspondingly compensated. On the other hand, apical URAT1 deletion significantly reduces urate reabsorption whereas ATP-binding cassette subfamily G member 2 (ABCG2) dysfunction affects uric acid excretion slightly that also help to explain the compensatory increase of FEur in severe renal dysfunction.

Some limitations of our study needed to be mentioned. The nature of cross-sectional study makes it hard to establish the causal relationship between eGFR and renal uric acid indicators. And the single urine collection may overestimate or underestimate the actual urinary excretion of uric acid. The clinical correlations also needed to be further clarified. A well-designed prospective study would be helpful for further studies to confirm the value of uric acid parameters. Nevertheless, our study is in line with the real clinical situation, in that many hyperuricemic patients have renal dysfunction. The strict sample collection and proper statistical methods further enhance the credibility of our study. And our results provided a new understanding of the value of uric acid indicators to better guide clinical treatment in CKD patients and provided clinical support for the study of underlying mechanisms.

## Conclusion

In conclusion, our study demonstrated that taking into account the special pathophysiological conditions of patients with CKD, Cur and Flur may be more reliable indicators of hyperuricemia classification although more evidence is needed to support this notion. On the other hand, uric acid may be excreted in other ways when renal function was severely declined, such as the intestinal excretion, suggesting treatment aimed at enhancing extra-renal excretion of uric acid may have clinical value in CKD patients with hyperuricemia.

## Data Availability

The datasets used and/or analyzed during the current study are available from the corresponding author on reasonable request.

## Abbreviations

CKD: Chronic kidney disease
eGFR: Estimated glomerular filtration rate
SUA: Serum uric acid
FEur: Fractional excretion of uric acid
EurGF: Excretion of uric acid per volume of glomerular filtration
UUA: Urinary uric acid excretion
Cur: Clearance of uric acid
FLur: Filtration load of uric acid
HB: Femoglobin
CRP: C-reactive protein
HbA1c: Hemoglobin A1c
ALB: Albumin
LDL: Low-density lipoprotein
HDL: High-density lipoprotein
TC: Total cholesterol
TG: Triglycerides
BUN: Blood urea nitrogen
Scr: Serum creatinine
BMI: Body mass index
Ucr: Urinary creatinine
Ccr: Clearance of creatinine
SNPs: Single nucleotide polymorphisms
